# Supplementary material for: Chemically specific termination control of oxide interfaces via layer-by-layer mean inner potential engineering
Source: Nat Commun. 2018 Jul 27;9:2965. doi: 10.1038/s41467-018-04903-4 (PMC6063925; doi:10.1038/s41467-018-04903-4)
Supplement: Supplementary file 1 — Supplementary Information [file 41467_2018_4903_MOESM1_ESM.pdf]

Chemically specific termination control of oxide interfaces via layer-by-layer mean inner potential engineering

Sun & Mao et al

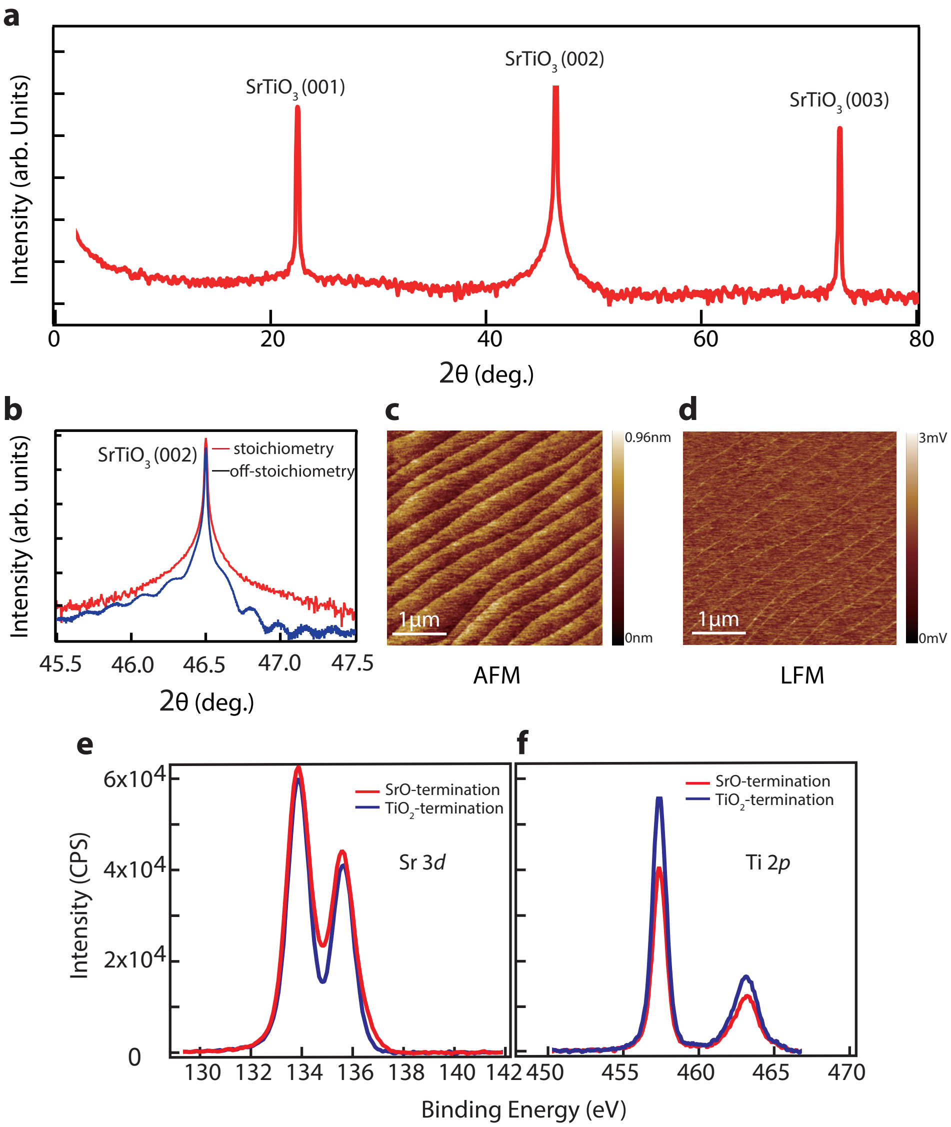

Supplementary Figure 1: ***Ex situ* characterizations of as-grown STO films.** (**a**), High-resolution θ-2θ XRD scan of 20 u.c. STO film with TiO_2-termination. (**b**), The fine scans around (002) STO peak of the stoichiometric and off-stoichiometric STO films. (**c**), AFM and (**d**), LFM images measured on the surface of the same sample in Supplementary Figure 1a. The XPS spectra of (**e**) Sr 3*d* and (**f**) Ti 2*p* peaks of 20 u.c. SrO-terminated STO film (red line), and TiO_2-terminated STO film (blue line).

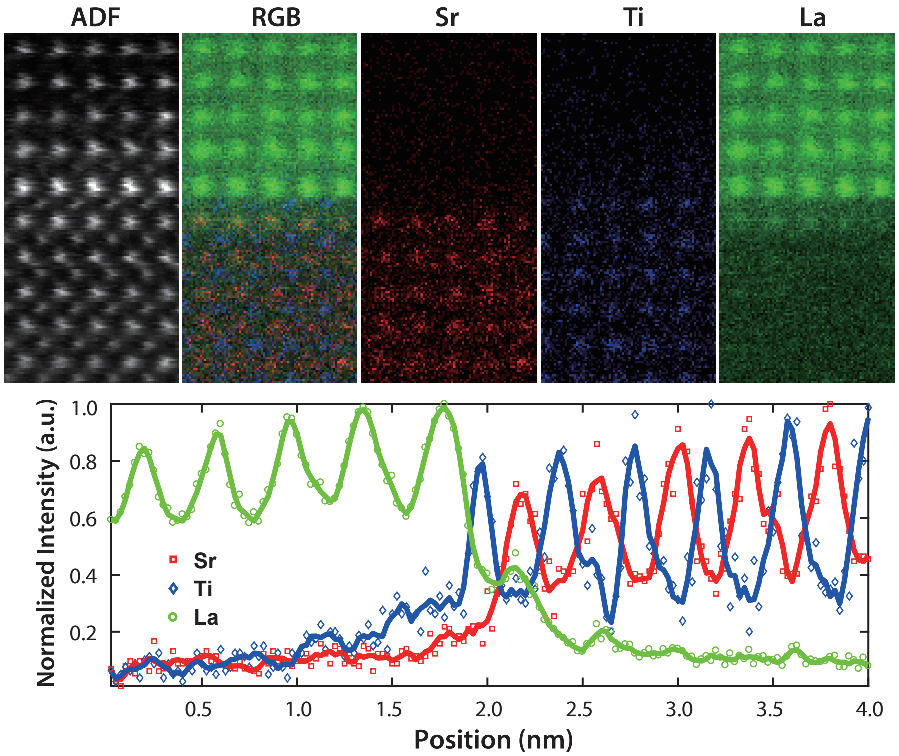

Supplementary Figure 2: The STEM-ADF images and elemental sensitive intensity analysis of the interface between 8 u.c. LAO and 20 u.c. STO film.

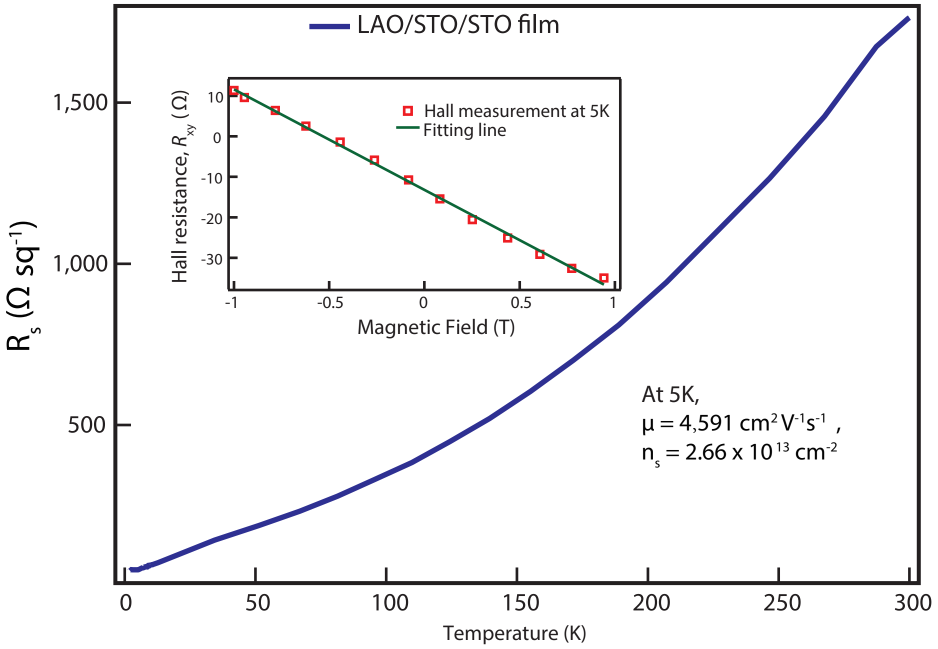

Supplementary Figure 3: **The transport measurements of the 2DEL at LAO (8 u.c.)/STO (20 u.c.) interface.** The inset is the corresponding Hall measurement applying a perpendicular magnetic field at low temperature.

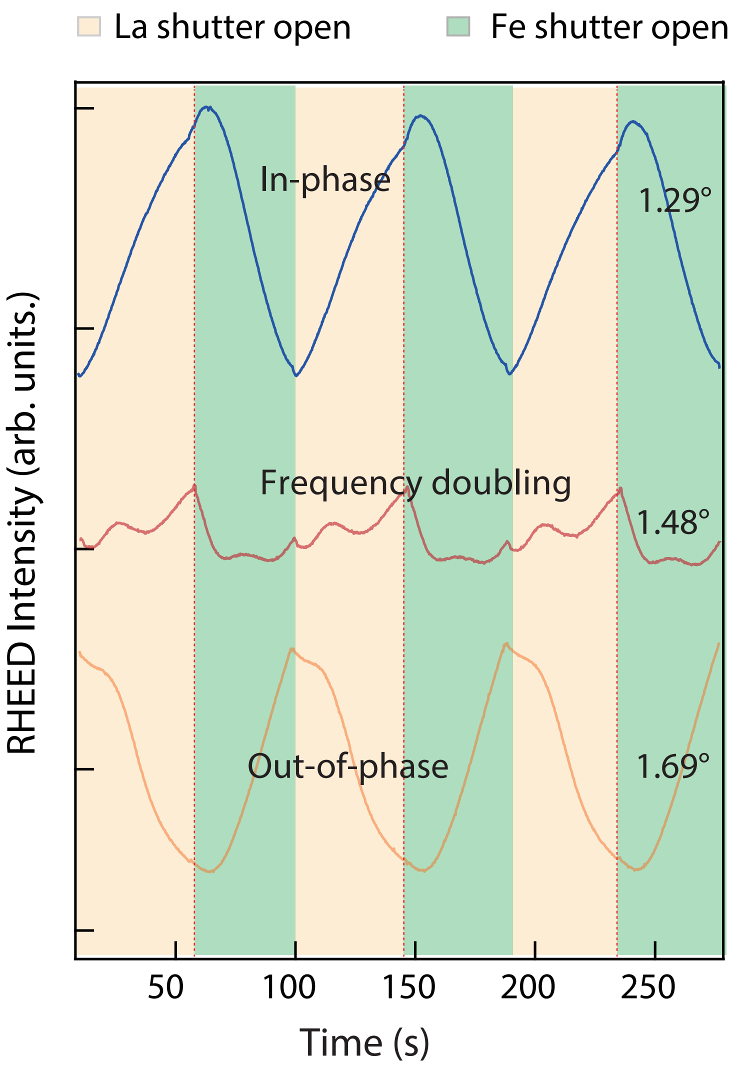

Supplementary Figure 4: Incident angle dependence of RHEED intensity oscillation curves in the shuttered-growth of $LaFeO_3$ films.

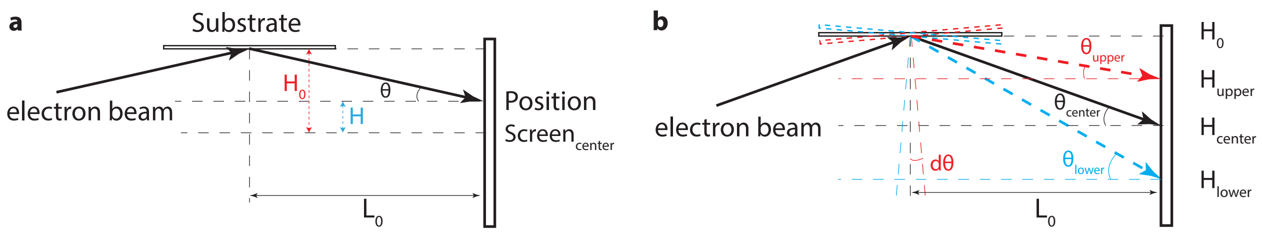

Supplementary Figure 5: Schematic of the electron beam incident angle calculations.

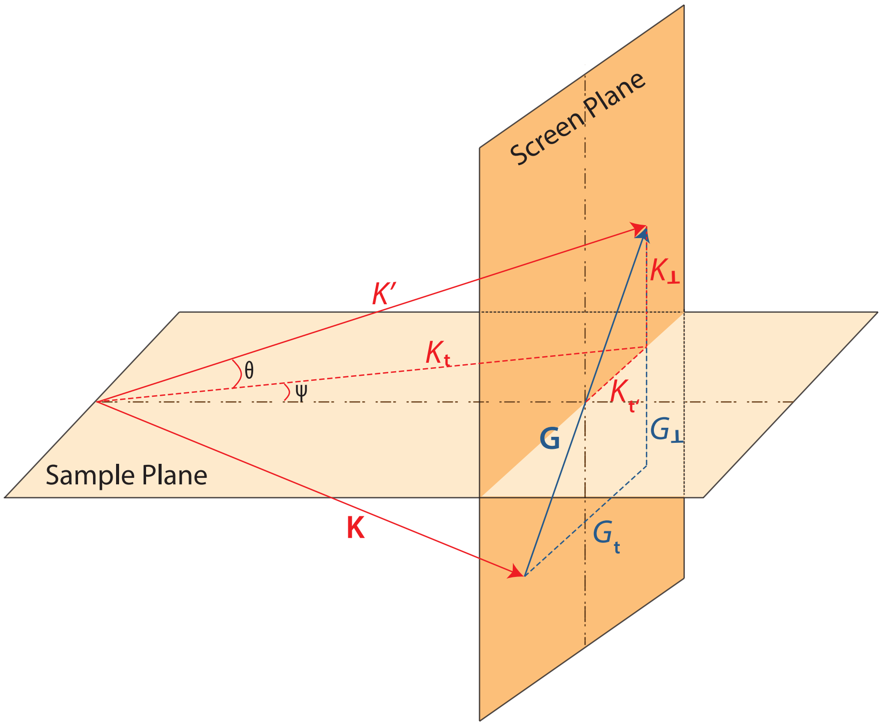

Supplementary Figure 6: **Schematic of the RHEED diffraction geometry.**

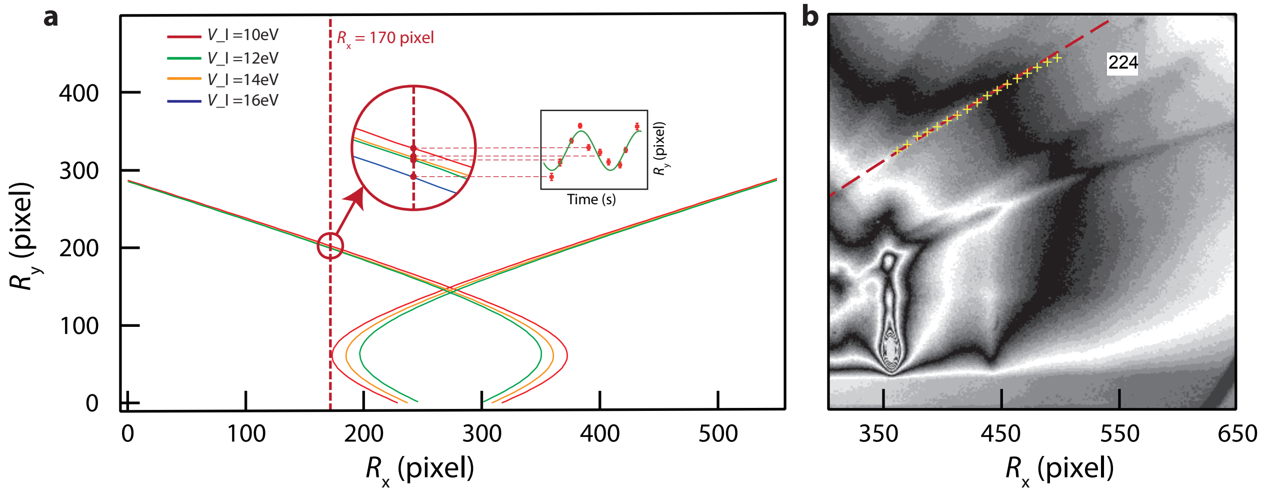

Supplementary Figure 7: Schematics of the position evolution of $\mathbf{R}_y$ as a function of mean inner potential $\mathbf{V}_I$ during growth and the origin of the systematic error of the mean inner potential due to the imperfect diffraction geometry.

Supplementary Note 1.
Ex situ characterizations of as-grown $SrTiO_3$ films

The $SrTiO_3$ (STO) films are characterized by x-ray diffraction with a Bruker D8 Discover diffractometer using Cu-K_α radiation in Supplementary Figure 1a. Long-range θ-2θ XRD scan of 20 u.c. TiO_2-terminated STO film shows no thickness fringes around all diffraction peaks and the FWHM of (002) is narrow, indicating the grown films are stoichiometric, homoepitaxial and cannot be distinguished from the STO substrate. Atomic force microscopy (AFM) image shows clear terraces and lateral force microscopy (LFM) measurement shows no contrast in Supplementary Figure 1c, indicating our as-grown films have the same high-quality TiO_2 termination as that of etched STO substrates.

At the same time, we fabricated both SrO- and TiO_2-terminated STO films for X-ray photoelectron spectroscopy (XPS) measurement. From Supplementary Fiure 1f, the extracted Sr $3d$/Ti $2p$ intensity ratio of SrO-terminated STO film and TiO_2-terminated STO film differs clearly and the values are 1.44 and 1.055 respectively. On the other hand, the theoretical calculation of the ratio from different terminations are 1.33 for SrO-terminated STO film and 1.12 for TiO_2-terminated STO film, roughly consistent with the experimental data. In the theoretical calculation, the total intensity for photoelectrons $I_j(\theta)$, is a weighted sum of the signals emanating from all sampling depths [1], the equation is shown as followed [2],

$$I_j(\theta) = K_j \sum_{i=0}^{\infty} n_{j,i} \exp\left(\frac{-Z_i}{\lambda_j \cos\theta}\right) \tag{1}$$

where K_j is a constant related to the elemental sensitivity and instrumental factors of atom j, which can be cancelled out in calculating the atomic concentrations and ratios. $n_{j,i}$ represents the atom fraction composition of element j in the ith layer, Z_i is the depth of the ith layer from the surface, λ_j is the characteristic attenuation length for the photoelectrons originating from element j and θ is the takeoff angle of emission measured from the sample normal, here $\theta = 0$. The characteristic attenuation length λ_j for the photoelectrons originating from Sr $3d$ and Ti $2p$ are calculated to be 25.69 $\AA$ and 20.77 $\AA$, respectively, using the NIST Electron Effective-Attenuation-Length database [3].

Based on the TiO_2-terminated STO film, we can fabricate the metallic interface by capping it with LAO films. The atomically abrupt interface between 8 u.c. LAO and 20 u.c. TiO_2-terminated STO film was confirmed by cross-section scanning transmission electron microscopy (STEM), in Supplementary Figure 2, STEM-annular dark-field (ADF) images were performed using JEOL JEM ARM 200F equipped with a cold field emission gun and an ASCOR fifth-order probe corrector. Simultaneous spectrum imaging of electron energy-loss spectroscopy (EELS) and energy-dispersive X-ray spectroscopy (EDS) was carried out under 200 kV accelerating voltage with a 26 mrad convergence angle for the optimal probe condition. Energy dispersion of 0.25 eV per channel and 91 mrad collection angle were set up for EELS and double large solid-state detectors from JEOL were used for EDS. The ADF image was acquired with a 93 mrad inner angle simultaneously. The Sr and Ti intensity maps were extracted from the EDS spectrum image by integrating across energy windows of 1.69-1.96 keV and 4.42-4.56 keV, respectively, while the La intensity map was extracted from the EELS spectrum image by integrating across the energy window of 829-860 eV. The Sr intensity map is from its L-α and the Ti map is from its K-α (EDS) while La map from its $L_{2,3}$ edges (EELS). The atomic-scale microstructure of the chemically abrupt interface further verifies the high-quality and the pure TiO_2 surface termination of STO film which is comparable with the previously reported articles [4–7]. The little diffusion of La atoms into SrO layer at the interface can be analyzed by the high growth temperature of LAO film.

Supplementary Note 2.
Transport measurements at the interface of $LaAlO_3/SrTiO_3$ films

The interfacial electronic properties of the $LaAlO_3/SrTiO_3$ (LAO/STO) heterostructure were probed using four-point electrical transport measurements (Quantum Design, Physical Properties Measurement System) using the Van der Pauw geometry. In Supplementary Figure 3, sheet resistance as a function of temperature reveals the n-type conducting interface between 8 u.c. LAO film and 20 u.c. STO film, showing a residual resistivity ratio (resistance

at 300K/resistance at 5K) of the order of 35. The inset picture shows the Hall measurement result, indicating the electron-dominated carriers as well as the high mobility up to 4,591 $cm^2V^{-1}s^{-1}$ at 5K, with an normal sheet carrier density of 2.66×10^{13} cm^{-2}, which is comparable with the literature [4, 8, 9].

Supplementary Note 3.
Phase inversion and frequency doubling in the growth of $LaFeO_3$ films

In Supplementary Figure 4, we show the angle dependence of RHEED oscillations in the shuttered-growth of $LaFeO_3$. Clear in-phase and out-of-phase oscillation patterns are observed and the frequency doubling is also exhibited although its quality is not as high as that in the growth of $SrTiO_3$ films. This indicates that the phase inversion and frequency doubling is an universal incident angle dependent phenomenon in the shuttered-growth of other perovskite oxides, regardless of the element species and polarity of the crystalline structure.

Supplementary Note 4.
Calculations of the electron beam incident angle

As shown in Supplementary Figure 5, the magnitude of the incident angle of the electron beam is determined by simple trigonometry calculations using the specular diffraction spots, such as the (11) diffraction spots in this case. In the measurements, the (11) and (-11) spots are tuned to be symmetric for simplicity. The incident angle θ of the electron beam is calculated as $\tan\theta = (H_0 - H)/L_0$, where H_0 and L_0 mean the vertical and horizontal distance between the substrate and the screen center respectively. H is the vertical distance between the (11) diffraction spot and the screen center, which can be extracted from RHEED patterns. In most cases, there is small tilting angle of the film along [001] axis and this deviation should be counted property to calculate correct incident angle. To account for this deviation, we rotate the sample by 180° around its normal axis, which shifts the diffraction spots up and down on the screen. As such, we use a simple correction formula to calculate incident angle, as follows,

At upper position: $\theta_{incident} = \theta_{center} - d\theta = \theta_{upper} - (\theta_{upper} - \theta_{lower})/4 = (3\theta_{upper} + \theta_{lower})/4$

At lower position: $\theta_{incident} = \theta_{center} + d\theta = \theta_{lower} + (\theta_{upper} - \theta_{lower})/4 = (\theta_{upper} + 3\theta_{lower})/4$

Supplementary Note 5.
RHEED intensity simulations

As described in the main text, we keep our discussion within the framework of kinematic diffraction theory since we mainly concentrate on the shift of the incident angle that satisfies the diffraction condition due to the mean inner potential variation and the resulting *relative* change of the diffraction intensity. The diffraction intensity is equal to the square of the diffracted amplitude $\mathbf{A}(\mathbf{s})$,

$$I(\mathbf{s}) = |\mathbf{A}(\mathbf{s})|^2 \tag{2}$$

And the diffracted amplitude is given as,

$$\mathbf{A}(\mathbf{s}) = \sum_j f_j(\mathbf{s}) \exp(-i\mathbf{s} \cdot \mathbf{r}_j) \tag{3}$$

where $f_j(\mathbf{s})$ is the atomic scattering factor, defined as,

$$f_j(\mathbf{s}) = \iiint \rho(\mathbf{r}) \exp(-i\mathbf{s} \cdot \mathbf{r})\, d\tau \tag{4}$$

where $\rho(\mathbf{r})$ is the atomic electron density, $\mathbf{r}$ means the lattice vector of the atoms in the film. And $\mathbf{s}$ is the scattering vector of the beam inside the crystal, defined as $\mathbf{s} = \mathbf{k'} - \mathbf{k}$, the wave vector $\mathbf{k}$ and $\mathbf{k'}$ correspond to the incident and

scattered electrons respectively, satisfying,

$$\mathbf{k}^2 = \mathbf{k'}^2 = 2m_e \left(E_0 + eV_{\mathrm{I}}\right)/\hbar^2 \tag{5}$$

where m_{e} is the mass of electron, V_{I} is the inner potential, E_0 is the energy of an electron in the vacuum above the surface.

For atomic scattering factor, when scattering vector $\mathbf{s} = 0$, f_j equals to the atomic number Z (Sr is 38, Ti is 22 and O is 8) [10].

When electrons enter a crystal, they will be scattered inelastically by both atoms and electrons, and the intensity of inelastic scattering is treated as an exponential reduction in that of the elastically scattered electrons. The attenuation factor is given by $\exp\left(-\mu_0 l\right)$ over a path length l. We measure the specular beam at a glancing angle θ (an internal angle), so l is twice of the path length to a layer jd below the surface, defined as $l = 2jd/\sin(\theta)$ and where d is the interplanar distance, j is the number of the layer. As a result, the diffraction intensity becomes,

$$I = I(\mathbf{s}) \exp\left(-2\mu_0 jd/\sin\theta\right) \tag{6}$$

where $I(\mathbf{s})$ is the elastic diffraction intensity.

Besides, we describe the surface as a two-dimensional Bravais mesh and separate the scattering vectors and lattice vectors into perpendicular and parallel components. Because the total energy and momentum are conserved at the direction parallel to the surface. Thus, we only consider the perpendicular direction. For infinite two-dimensional lattices, the summation of diffracted intensity becomes,

$$I(s_{\mathrm{z}}) = \left| N_{mesh} \sum_{j \in mesh} f_j \exp\left(-i s_{\mathrm{z}} z_j\right) \right|^2 \tag{7}$$

where s_{z} and z_j is the perpendicular component of $\mathbf{s}$ and $\mathbf{r}$ respectively, and we have put $N_{mesh} = 1$ for simplicity. When consider the attenuation factor, the diffraction intensity is calculated by the equation below,

$$I = \left| \sum_{j \in mesh} f_j \exp\left(-i s_{\mathrm{z}} z_j\right) \cdot \exp\left(-\mu_0 jd/\sin\theta\right) \right|^2 \tag{8}$$

Assuming there is no surface relaxation, j refers to the number of layer below the surface, then put $z_j = -jd$ $(j = 0, 1, 2, \cdots)$

Note that the perpendicular momentum transfer corresponding to internal angle is given as $s_{\mathrm{z}} = 2\sqrt{K_{\mathrm{z}}^2 + U}$, where $K_{\mathrm{z}} = K \sin\theta$, which is the perpendicular component of the incident wave vector in the vacuum, satisfying $K = \sqrt{2m_e E_0}/\hbar$, and U is related to the mean inner potential V_{I}, given as $U = (2m_e e/\hbar^2)V_{\mathrm{I}}$. (As mentioned before, V_{I} for SrO-(TiO_2-) terminated surface is 13.3 V(14.6 V))

In our simulations, μ_0 is the absorption coefficient calculated as the reciprocal of the mean free path L for electrons in the vacuum, $E_0 = 15$ keV, $L = 110$ Å, $n = 200$, $e \doteq 1.602 \times 10^{-19}$ C, so the final diffraction intensity simulation function we used is,

$$I = \left| f_j \sum_{j=0}^{\infty} \exp\left(i2\sqrt{(2m_e/\hbar^2)E(\sin\theta)^2 + (2m_e e/\hbar^2)V_{\mathrm{I}}} \cdot jd \right) \cdot \exp\left(-jd/(L\sin\theta)\right) \right|^2 \tag{9}$$

When performing the simulations, we separate the $SrTiO_3$ film into two parts, SrO layers and TiO_2 layers, in the direction perpendicular to the surface. In the case of SrO-termination, the first layer is SrO layer, $j = 0$, the second layer is TiO_2 layer, $j = 1$, and so on, thus, interplanar distance is half of the lattice constanct a, that's to say, $d = a/2 = 3.905/2 = 1.9525$ Å. For different layers, the atomic scattering factor used is different. Also, we want to reveal the relationship between the diffraction intensity inside the STO film and the external angle instead of the internal angle, so the following equation is used during the calculation.

$$\theta_{ext} = \sqrt{\theta_{in}^2 - eV_{\mathrm{I}}/E_0} \tag{10}$$

Supplementary Note 6.
Mean inner potential extracted from RHEED intensity vs incident angle

Kikuchi lines are formed by inelastically scattered electrons, which can be easily understood in the electron diffraction scheme. In some directions, these inelastically scattered electrons will satisfy the Bragg condition for a set of lattice planes (hkl), then form Kikuchi lines. Thus, the Kikuchi lines for the set of planes corresponding to the reciprocal lattice vector $\mathbf{G}(hkl)$ is given by

$$\mathbf{k} \cdot \mathbf{G} = \frac{|G|^2}{2} \tag{11}$$

or

$$\mathbf{K}_{\mathrm{t}} \cdot \mathbf{G}_{\mathrm{t}} + \left(\mathrm{K}_{\perp}^2 + U\right)^{1/2} \mathrm{G}_{\perp} = \frac{|G|^2}{2} \tag{12}$$

where $\mathbf{G}$ is the reciprocal lattice vector, U is given as $U = (2m_e e/\hbar^2)V_{\mathrm{I}}$, where V_{I} is the mean inner potential of the crystal. $\mathbf{G}_{\mathrm{t}}$ ($\mathbf{G}_{\perp}$) and $\mathbf{K}_{\mathrm{t}}$ ($\mathbf{K}_{\perp}$) are the surface parallel (normal) components of the reciprocal lattice vector and incident wave vector in the vacuum, respectively (see Supplementary Figure 6).

In our case, the electron beam is injected along the [1 1 l] direction and k=h, so that $\mathbf{G}_{\mathrm{t}} = h\mathbf{a}_1^* + k\mathbf{a}_2^* = h(\mathbf{a}_1^* + \mathbf{a}_2^*)$, which yields,

$$\sqrt{2}h\,|\mathbf{a}_1^*|\sin\Psi\cos\theta + l\,|\mathbf{a}_3^*|\left(\theta^2 + \frac{eV_{\mathrm{I}}}{E_0}\right)^{1/2} = \frac{\mathrm{G}^2}{2K},$$

As θ and Ψ are small in RHEED measurements, $\lim_{\theta\to 0}\sin\theta = \theta$ and $\lim_{\Psi\to 0}\sin\Psi = \Psi$, the above equation becomes,

$$\sqrt{2}h\,|\mathbf{a}_1^*|\,\Psi\cos\theta + l\,|\mathbf{a}_3^*|\left(\theta^2 + \frac{eV_{\mathrm{I}}}{E_0}\right)^{1/2} = \frac{\mathrm{G}^2}{2K},$$

where $K = \sqrt{2m_{\mathrm{e}}E_0}/\hbar$, Ψ is the complementary angle of included angle between $\mathbf{K}_{\mathrm{t}}$ and screen normal. For $SrTiO_3$, $\mathbf{a}_1^* = \mathbf{a}_3^* = 2\pi/a$, $a = 3.905$ $\AA$ and $|G|^2 = (8\pi^2h^2 + 4\pi^2l^2)/a^2$.

Thus, we obtain a relationship between Ψ and θ,

$$\Psi = \frac{\frac{\mathrm{G}^2}{2K} - l\,|\mathbf{a}_3^*|\left(\theta^2 + \frac{eV_{\mathrm{I}}}{E_0}\right)^{1/2}}{\sqrt{2}h\,|\mathbf{a}_1^*|\cos\theta} \tag{13}$$

Since the RHEED spectra are screenshot images, we need to further convert the angle Ψ and θ into the position axises of R_{y} and R_{x} according to

$$\begin{aligned}\Psi &= (R_{\mathrm{x}} - R_{\mathrm{x0}})/L_0 \\ \theta &= (R_{\mathrm{y}} - R_{\mathrm{y0}})/L_0\end{aligned} \tag{14}$$

Where L_0 is the distance between the center of sample and the screen, which is 290 mm in our case. R_{x0} and R_{y0} are the position of the projection of the sample centre on the screen. Substituting Equation (14) into Equation (13), yields

$$R_{\mathrm{x}} = \frac{L_0(\frac{\mathrm{G}^2}{2K} - l\,|\mathbf{a}_3^*|\left((\frac{R_{\mathrm{y}}-R_{\mathrm{y0}}}{L_0})^2 + \frac{eV_{\mathrm{I}}}{E_0}\right)^{1/2})}{\sqrt{2}h\,|\mathbf{a}_1^*|\cos\frac{R_{\mathrm{y}}-R_{\mathrm{y0}}}{L_0}} + R_{\mathrm{x0}} \tag{15}$$

In a RHEED spectrum, R_{x0}, R_{y0} are the same for all Kikuchi lines. By fitting two or more Kikuchi lines, R_{x0}, R_{y0}, V_{I} can be extracted.

In our work, we take many RHEED spectra during the shutter-controlled layer-by-layer growth of $SrTiO_3$ epitaxial

films. For each spectrum, we extract the mean inner potential (V_I) by fitting the Kikuchi lines corresponding to $\mathbf{G}$(2,2,4) and $\mathbf{G}$(1,1,3) using Equation (15). As shown in Fig. 2 in the main text, we observe a clear termination dependence variation of the mean inner potential in the growth of $SrTiO_3$. This is the first demonstration of *in situ* measurements of the mean inner potential during the growth of oxide films in real time.

Note that the termination-dependent position shift of the Kikuchi lines are small and a stable RHEED system is needed for good data fitting of the Kikuchi lines. Also, clear and sharp Kikuchi lines from high quality film growth are needed for reliable data analysis.

Supplementary Note 7.
Absolute and relative mean inner potential

By fitting the Kikuchi lines using Equation (15), the extracted absolute value of the mean inner potential shows periodic oscillations during the growth, but the fitting error bar is relatively large (Figure 2c and Supplementary Figure 7b). This is because the lineshape of the real Kikuchi lines can not be perfectly accounted by ideal equation due to the imperfect diffraction geometry and the distortion of RHEED images taken by the CCD camera. For example, RHEED screen is not perfectly perpendicular to the electron beam. Nonetheless, the position of the Kikuchi lines also oscillates during the growth and the small fitting error bar indicates the relative change of the mean inner potential is clear and reliable (Figure 2c and Supplementary Figure 7a).

Supplementary References

[1] Livesey, A. K. & Smith, G. C. The determination of depth profiles from angle-dependent xps using maximum entropy data analysis. *J. Electron. Spectrosc.* **67**, 439–461 (1994).
[2] Nie, Y. F. *et al.* Atomically precise interfaces from non-stoichiometric deposition. *Nat. Commun.* **5**, 4530 (2014).
[3] Powell, C. J. & Jablonski, A. Nist electron effective-attenuation-length database - version 1.1. *J. Surf. Anal.* **9**, 322–325 (2002).
[4] Warusawithana, M. P. *et al.* $LaAlO_3$ stoichiometry is key to electron liquid formation at $LaAlO_3/SrTiO_3$ interfaces. *Nat. Commun.* **4**, 2351 (2013).
[5] Lee, P. W. *et al.* Hidden lattice instabilities as origin of the conductive interface between insulating $LaAlO_3$ and $SrTiO_3$. *Nat.Commun.* **7**, 12773 (2016).
[6] Lee, H. *et al.* Direct observation of a two-dimensional hole gas at oxide interfaces. *Nat. Mater.* (2018).
[7] Nakagawa, N., Hwang, H. Y. & Muller, D. A. Why some interfaces cannot be sharp. *Nat. Mater.* **5**, 204–209 (2006).
[8] Fte, A. *et al.* Growth-induced electron mobility enhancement at the $LaAlO_3/SrTiO_3$ interface. *Appl. Phys. Lett.* **106**, 1601 (2015).
[9] Bell, C., Harashima, S., Hikita, Y. & Hwang, H. Y. Thickness dependence of the mobility at the $LaAlO_3/SrTiO_3$ interface. *Appl. Phys. Lett.* **94**, 423 (2009).
[10] Ichimiya, A. & Cohen, P. I. *Reflection high-energy electron diffraction* (Cambridge University Press, 2004).
